# Poly(Acrylic Acid)-Sodium Alginate Superabsorbent Hydrogels Synthesized by Electron Beam Irradiation Part I: Impact of Initiator Concentration and Irradiation Dose on Structure, Network Parameters and Swelling Properties

**DOI:** 10.3390/ma16134552

**Published:** 2023-06-23

**Authors:** Gabriela Craciun, Ion Cosmin Calina, Maria Demeter, Anca Scarisoreanu, Marius Dumitru, Elena Manaila

**Affiliations:** Electron Accelerators Laboratory, National Institute for Laser, Plasma and Radiation Physics, 409 Atomistilor St., 077125 Magurele, Romania; gabriela.craciun@inflpr.ro (G.C.); calina.cosmin@inflpr.ro (I.C.C.); maria.dumitrascu@inflpr.ro (M.D.); anca.scarisoreanu@inflpr.ro (A.S.); marius.dumitru@inflpr.ro (M.D.)

**Keywords:** sodium alginate, acrylic acid, hydrogels, poly(ethylene oxide), potassium persulphate, electron beam irradiation

## Abstract

In the present paper, hydrogels based on acrylic acid (20%), sodium alginate (0.5%) and poly(ethylene oxide) (0.1%) were obtained by electron beam irradiation at room temperature with doses between 5 and 20 kGy, using potassium persulfate in concentrations up to 0.3% as a reaction initiator. The influence of initiator concentration and irradiation dose on hydrogel network parameters, swelling and deswelling behavior, gelation and degradation points, structure and morphology were investigated. Cross-link density increased with the irradiation dose and initiator addition, except at 20 kGy. The gel fraction was over 87.0% in all cases. Swelling experiments in distilled water showed swelling degrees of 40,000% at an irradiation dose of 5 kGy when a concentration of 0.1% initiator was added. A relationship between the swelling degree and irradiation dose, cross-linking degree (that increases from 0.044 × 10^2^ to 0.995 × 10^2^ mol/cm^3^) and mesh size (that decreases from about 220 nm to 26 nm) was observed. The addition of only 0.1% of PP led to the obtaining of hydrogels with a swelling degree of 42,954% (about 430 g/g) at an irradiation dose of 5 kGy and of 7206% (about 62 g/g) at 20 kGy, which are higher percentages than those obtained in the same irradiation conditions but without PP.

## 1. Introduction

Hydrogels, 3D polymeric water-insoluble networks that can absorb large amounts of water compared with their weights [1,2,3,4,5], are extremely attractive polymeric materials for the medical, pharmaceutical, food, and agriculture fields [6]. In the medical and pharmaceutical fields, hydrogels can be used as a support with the role of mechanical protection of tissues, in which the cells are suspended or attached to the polymeric material [6,7,8], and in the food industry for the encapsulation of different active ingredients [9].

The use of superabsorbent hydrogels is a common method of water conservation to increase production in agriculture [1,2,3,4,5]. These materials may also serve as vehicles for different soil conditioners or agrochemicals because they can regulate their release rate [10].

Hydrogels can be classified depending on their physical properties (smart or conventional), source (natural, synthetic, or hybrid), ionic charges (cationic, anionic, or non-ionic), rate of biodegradation (biodegradable or non-biodegradable), nature of cross-linking (physically or chemically cross-linked), chemical response (pH, glucose, or oxidant-responsive), physical response (temperature, pressure, light, electric, or magnetic field-responsive), and preparation method (copolymeric, homopolymeric, or interpenetrating networks) [11].

In addition to physical and chemical cross-linking, the obtaining methods based on ionizing and non-ionizing radiation are more and more attractive due to their simplicity, hydrogels’ special properties and stability, lack of residual toxic substances, and not least, due to their low cost and energy consumption [12,13]. In particular, electron beam irradiation offers advantages over other ionizing radiation sources of easy process control, the possibility of joining hydrogel formation and sterilization in one technological step, low concentrations of initiators, cross-linkers or other additives, no waste, relatively low running costs, and sometimes more structural homogeneity of the polymer network [14,15]. There are many ways to obtain hydrogels by radiation-based techniques, including irradiation of solid polymers, monomers (in bulk or solution), or aqueous solutions of polymers [15].

Most of the currently used hydrogels are based on derivatives of poly(acrylamide) and acrylate, so they are not completely biodegradable and are considered potential sources of contaminants for soil and plants due to a certain degree of toxicity of monomers [10]. For these reasons, the development of biodegradable hydrogels for use in agriculture is of high interest worldwide. Low-cost biopolymers such as alginate, chitosan, cellulose, and their derivatives are being explored due to their biocompatibility and biodegradability [10].

Polysaccharides are among the most widely used hydrophilic polymers for hydrogel preparation due to their many benefits compared to synthetic polymers. Some of the fields of application for these cross-linked polymers are pharmacy, chemical engineering, agriculture, and food [16].

Sodium alginate, a natural polysaccharide derived from brown seaweeds, is composed of D-mannuronic acid and D-guluronic acid [17,18,19,20,21]. Due to its biodegradability, it has been used extensively in drug delivery applications, but recent studies cite many applications of sodium alginate in agriculture in a cross-linked or grafted state [17,18,22,23]. For different biomedical applications, especially drug delivery systems, hydrogels based on sodium alginate and poly(ethylene oxide) (PEO) have been obtained, especially by the solution casting method. As it is a very good delivery vehicle, non-toxic, and water-soluble, PEO may be used as a constituent in hydrogels for agriculture. PEO is a suitable candidate to be blended with sodium alginate because the hydroxyl groups from sodium alginate may form hydrogen bonds with ether oxygen from PEO and because the miscibility of polymer blends is mainly determined by the chemical structure, composition, and molecular mass of each component [24,25]. PEO addition to cross-linked hydrogels by radiation techniques improves their physical and mechanical properties and makes them suitable for application as, for example, wound dressings [26].

In the present paper, hydrogels based on acrylic acid (20%), sodium alginate (0.5%), and poly(ethylene oxide) (0.1%) were obtained by electron beam irradiation at room temperature with irradiation doses between 5 and 20 kGy. Potassium persulfate in concentrations up to 0.3% was used as a reaction initiator. The influence of initiator concentration and irradiation dose on hydrogels’ structure, morphology, and network parameters was investigated. Swelling and deswelling behavior were investigated in distilled water. The experiment’s organization and the results obtained will contribute to the advancement of knowledge about the subject.

## 2. Materials and Methods

### 2.1. Materials

Sodium alginate (SA, M_w_ = 120,000–190,000 g/mol, viscosity = 15–25 cP, 1% in water, acrylic acid (AA, M_w_ = 71.08 g/mol, density = 1.13 g/cm^3^), poly(ethylene oxide) (PEO, Mw = 300,000 g/mol, density = 1.210 g/cm^3^) and potassium persulfate (PP, M_w_ = 270.322 g/mol, density = 2.477 g/cm^3^) have been purchased from Merck KGaA, Darmstadt, Germany and used for hydrogels obtaining without any further modification.

### 2.2. Experimental Installation and Samples Preparation

Poly(acrylic acid)-sodium alginate hydrogels have been obtained by electron beam irradiation, under a rigorous control of dose and dose rate, using the linear accelerator of 5.5 MeV, ALID 7, built in the Electron Accelerators Laboratory from the National Institute for Lasers, Plasma and Radiation Physics, Bucharest, Romania. Radiation dosimetry was performed using a graphite calorimeter which is the primary standard for electron beams.

Solutions with fixed concentrations of SA (0.5%), AA (20%), PEO (0.1%), and variable concentrations of PP (between 0 and 0.3%) have been placed in medical syringes with a diameter of 15 mm and irradiated with e-beam in atmospheric conditions and room temperature of 25 °C. The irradiation doses were between 5 and 20 kGy, and the dose rate was 0.9 kGy/min. The obtained hydrogels have been kept at room temperature for 24 h, then removed from the syringes in cylindrical form and prepared for analysis by cutting into discs of 3–4 mm height, washing them with ethanol for unreacted chemicals removal, drying them in the air for three days, then in a laboratory oven at 50 °C for 24 h to reach a constant weight, and stored in desiccators.

### 2.3. Hydrogels Characterization

#### 2.3.1. Measurements of Gel Fraction, Swelling, and Network Parameters

Dried samples of 0.2 g (*W_i_*) have been immersed in distilled water for 72 h at room temperature for soluble fraction removal, dried in air for 6 days, and then in a vacuum oven for 48 h at 50 °C to constant weight (*W_f_*). The gel fraction (*G*) and sol fraction (*s*) were determined based on Equations (1) and (2) respectively [27].

(1) $$G(\%)=\frac{W_{f}}{W_{i}}\times 100,$$

(2) s=1−G,

The swelling dynamics and water uptake at equilibrium have been determined by swelling experiments in distilled water at a room temperature of 25 °C. The mass increase was evaluated by regular weighing (the weight *W_t_* determined at time *t*) until the equilibrium has been reached. The swelling *S*(%) and swelling at equilibrium *S_eq_.*(%) have been calculated using Equations (3) and (4) [28,29].

(3) $$S(\%)=\frac{W_{t}-W_{i}}{W_{i}}\times 100,$$

(4) $$S_{eq.}(\%)=\frac{W_{eq.}-W_{i}}{W_{i}}\times 100,$$

The cross-link density, or mole fraction, of the cross-linked units (*q*) has been calculated using Equations (5)–(7) [29,30,31,32] as being the ratio between the average molar mass between cross-links determined according to the theory of Flory and Rehner (*M_c_*) and the molecular weight of the repeating units from polymer (*M_r_*).

(5) $$q=\frac{M_{c}}{M_{r}},$$

(6) $$M_{C}=-V_{s}d_{P}\frac{\nu_{2,S}^{1/3}-\nu_{2,S}/2}{\ln(1-\nu_{2,S})+\nu_{2,S}+\chi \nu_{2,S}^{2}},$$

(7) $$M_{r}=\frac{\left(m_{SA}\times M_{SA}\right)+\left(m_{AA}\times M_{AA}\right)+\left(m_{PEO}\times M_{PEO}\right)}{m_{SA}+m_{AA}+m_{PEO}},$$

where *V_s_* is the molar volume of water (18 cm^3^ mol^−1^); *d_P_* is the polymer density; *υ_2,s_* (cm^3^) is the volume fraction of the polymer in the swollen gel; *χ* is the Flory–Huggins interaction parameter between the solvent and polymer; *m_SA_* (g) is the mass of SA; *m_AA_* (g) is the mass of AA; *m_PEO_* (g) is the mass of PEO; *M_SA_* (g mol^−1^*)* is the molar mass of SA; *M_AA_* (g mol^−1^*)* is the molar mass of AA; *M_PEO_* (g mol^−1^*)* is the molar mass of PEO.

The Flory–Huggins interaction parameter between the solvent and polymer, *χ*, has been determined using Equation (8), and the volume fraction of the polymer in the swollen gel (*υ*_2*,s*_) using Equation (9).

(8) $$\chi =\frac{\ln(1-\nu_{2,s})+\nu_{2,s}}{\nu_{2,s}^{2}},$$

(9) $$\nu_{2,s}={\left[1+\frac{d_{p}}{d_{s}}\left(\frac{W_{g}}{W_{i}}-1\right)\right]}^{-1},$$

where *d_P_* is the density of hydrogel; *d_s_* is the density of solvent; *W_g_* is the mass of the hydrogel in swelled form; *W_i_* is the initial mass of the dried gel.

Sample densities (between 1.045–1.285) have been determined using an analytical balance equipped with density kit tools (AS 220, 0.1 mg resolution, producer Radwag, Warsaw, Poland).

Two other important parameters in hydrogels characterization, mesh size (*ξ*) and porosity (*P*), have been determined using Equation (10) [33] and Equation (11), respectively [29]:

(10) $$\xi =\upsilon_{s}^{-1/3}l\sqrt{\frac{2C_{n}M_{c}}{M_{r}}},$$

(11) $$P(\%)=\frac{V_{d}}{1-V_{d}}\times 100,$$

where *υ_S_* is the volume fraction of the polymer in the swollen gel; *l* is the length of the C–C bond along the polymer backbone (0.154 nm); *C_n_* is the Flory characteristic ratio of the polymer; *M_c_* is the average molar mass between cross-links; *M_r_* is the molecular mass of the repeated unit, *V_d_* is the volume ratio of water at equilibrium.

The characteristic ratio *C_n_* was taken as the weighted average of *C_n_* values for poly(AA), SA, and PEO chains, according to their molar ratio in the hydrogel: poly(AA) = 6.7 [33], SA = 21.1 [34] and PEO = 4.98 [35].

The water retention capacity was determined by placing swollen hydrogels at equilibrium (Seq) in Petri dishes and keeping them in the air to study their deswelling. Changes in hydrogel mass or water retention were calculated by measuring the mass of each sample at regular time points:

(12) $$Deswelling=\frac{m_{t}}{m_{eq.}}\times 100,$$

where *m_eq._* and *m_t_* are the masses of hydrogels at equilibrium and at the time *t*, respectively [36].

#### 2.3.2. Sol-Gel Analysis

The free software Gelsol95, based on the Charlesby–Rosiak equation [37], was used to determine the gelation dose and degradation to the cross-linking ratios of the hydrogels.

(13) $$s+\sqrt{s}=\frac{p_{0}}{q_{0}}+\left(2-\frac{p_{0}}{q_{0}}\right)\left(\frac{D_{v}+D_{g}}{D_{v}+D}\right),$$

where *s* is the sol fraction; *p*_0_ is the degradation density; *q*_0_ is the cross-linking density; *D* (kGy) is the irradiation dose; *D_g_* (kGy) is the gelation dose; *D_V_* (kGy) is the virtual dose.

The radiation yields of cross-linking (*G_X_*) and degradation (*G_S_*) were calculated using Equations (14) and (15) [37]:

(14) $$G_{X}=\frac{4.9\times 10^{2}\times c}{M_{c}\times D\times \rho },$$

(15) $$G_{S}=G_{X}\times 2\frac{p_{0}}{q_{0}},$$

where *G_X_* is the number of moles of cross-linked bonds per Joule; *G_S_* (mol/J) is the radiation yield of degradation; *M_C_* (g/mol) is the average molecular weight between two successive cross-link points; *c* (g/L) is the polymer concentration in the irradiated solution; *D* (J/kg) is the absorbed dose; *ρ* (kg/m^3^) is the polymer density.

#### 2.3.3. Structural Investigations by Fourier-Transform Infrared Spectroscopy

The hydrogel structure has been investigated by the FTIR technique using the Spectrum 100 instrument (Perkin Elmer, Waltham, MA, USA). Spectra were collected in ATR mode at a resolution of 4 cm^–1^ in the range of 4000–650 cm^–1^, with 30 scans performed for each sample. All spectra were analyzed using Spectrum v. 6.3.2 software.

#### 2.3.4. Morphological Investigations by Scanning Electron Microscopy

Lyophilized hydrogels have been examined by Scanning Electron Microscopy (SEM) technique using the FEI/Phillips scanning electron microscope (Hillsboro, OR, USA).

## 3. Results and Discussion

During e-beam irradiation, free radicals are formed that induce either cross-linking or degradation processes. The recombination reaction of free radicals leads to the occurrence of the cross-linking process, while degradation occurs when the radical-radical reactions fail. The influence of irradiation dose and initiator concentration on the semi-synthetic hydrogels based on sodium alginate (SA) and acrylic acid (AA) has been tested. To increase the cross-linking effect and reinforce the hydrogel network, PEO was included in the hydrogel composition.

### 3.1. Gel Fraction and Network Parameters

**Figure 1:**
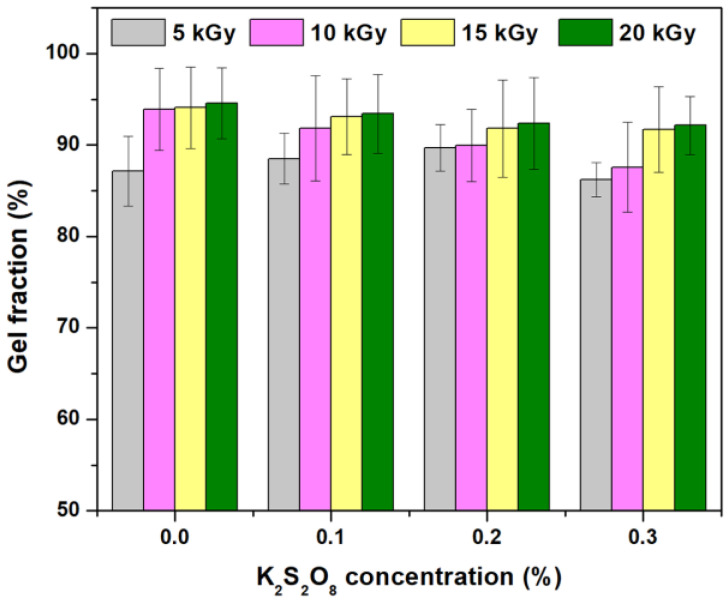
The effect of irradiation dose and PP concentration on gel fraction.

**Figure 2:**
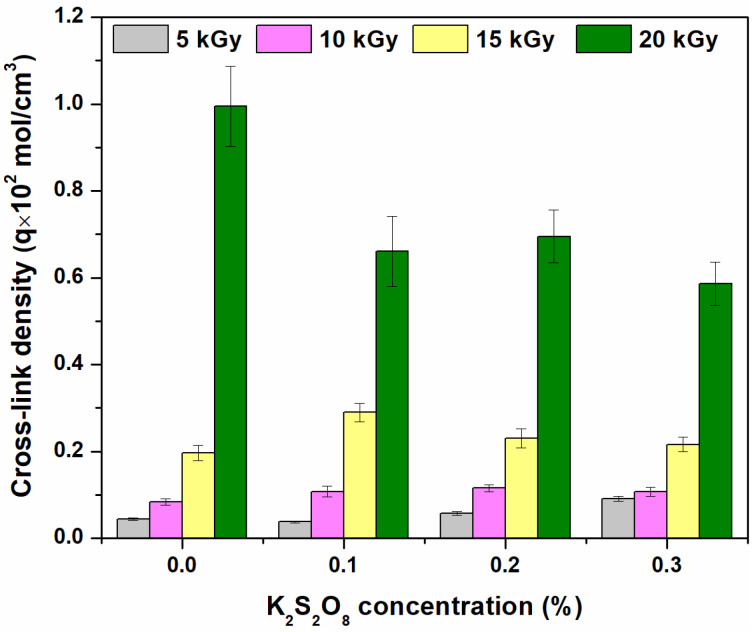
The effect of irradiation doses and PP concentration on cross-link density.

**Figure 3:**
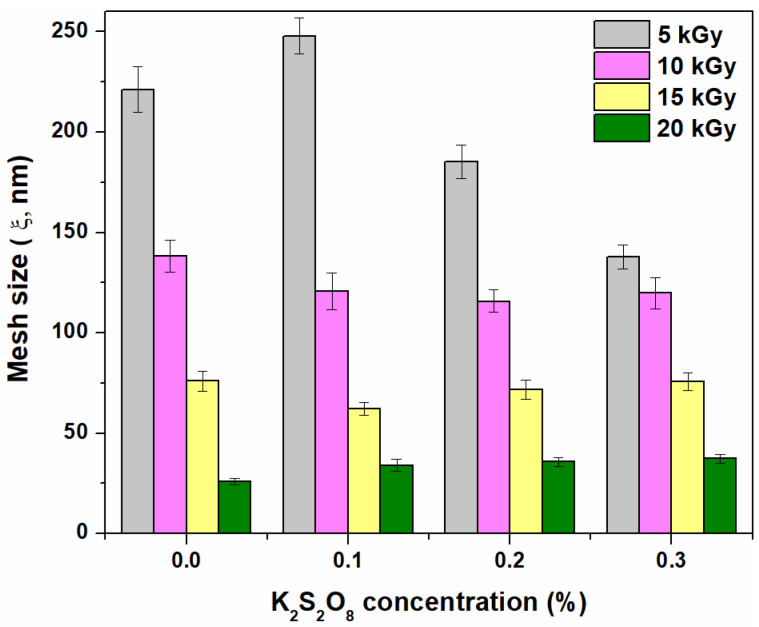
The effect of irradiation doses and PP concentration on mesh size.

The results regarding the behavior of gel fraction and network parameters as a function of irradiation dose and PP concentration are presented in Figure 1, Figure 2 and Figure 3 and Table 1.

As can be seen from Figure 1, the increase in irradiation dose has led to an increase in the hydrogel gel fraction from 87.1% to 94.6% without PP. Contrary to expectations, adding PP as an initiator has led to a gel fraction decrease that is lower at 20 kGy than those obtained without it: i.e., 93.4%, 92.4%, and 92.1% for 0.1%, 0.2%, and 0.3% PP, respectively. Both the gel fraction and cross-link density (Figure 2) are strongly influenced by the irradiation dose and the initiator concentration. As can be seen from Figure 2, the “hyper cross-linking” effect is more pronounced in the case of e-beam irradiation [38] and is usually associated with the mesh size decreasing. This behavior has also been observed at the transition from 15 to 20 kGy.

All investigated hydrogels have shown gel fractions over 87.0%, regardless of the irradiation dose. Hydrogels with a high gel fraction (>87.0%) can be obtained even at the lowest irradiation dose of 5 kGy, showing that grafting and cross-linking reactions take place even at low irradiation doses. Even if high values of gel fraction and cross-link density have been obtained in the absence of PP, the mesh size has decreased (Figure 3) and, as a consequence, the water absorption degree is expected to be affected. Furthermore, at low irradiation doses and in the absence of an initiator, brittle hydrogels at maximum swelling were obtained. The concentration of 0.1% PP has led to the obtaining of hydrogels with a gel fraction over >87.0%, as in the case of samples without PP, but more stable in the swollen state. Increasing the PP concentration to 0.2 and 0.3%, respectively, led to stable hydrogels with smaller mesh sizes. From Table 1, it is observed that the molecular weight between two successive cross-linking points (Mc) decreased with increasing irradiation dose, regardless of the presence or absence of PP. For this reason, the variations due to the addition of PP are almost irrelevant.

Regarding the polymer volume fraction in the swollen gel (υ_2,s_), its behavior was opposite to that of *Mc* as the irradiation dose increased. The υ_2,s_ value increased with the irradiation dose and showed insignificant variations with increasing PP concentration. From Table 1, it can be seen that all hydrogels present porosities of >98.0%.

It is well-known that cross-linking, polymerization, grafting, and degradation reactions compete during the irradiation process. 

The mixture that formed the basis of the hydrogel synthesis contains SA, AA, and PEO. Following e-beam irradiation, a polymeric network was formed due to the chemical cross-linking of AA, mainly due to its grafting on the SA and PEO chains, but also due to the physical cross-linking of SA [39,40]. 

The addition of PEO was conducted to strengthen the hydrogel network and improve its stability after reaching the maximum degree of absorption [41]. As a natural polysaccharide, SA is the salt form of alginic acid and degrades when exposed to irradiation doses higher than 20 kGy due to breakage of the main chains [39,42]. The presence of potassium persulfate (PP) can lead to the acceleration of the SA degradation process with an increase in the irradiation dose [43].

**Figure 4:**
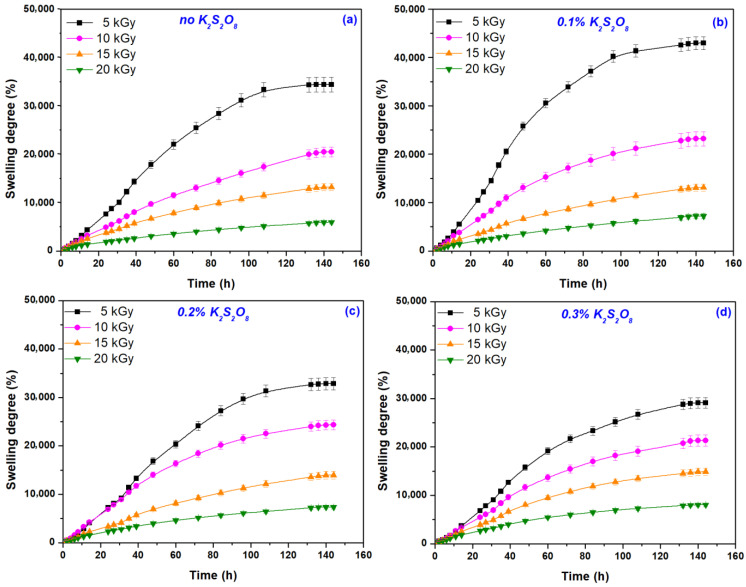
The swelling degree of hydrogels in distilled water depending on the irradiation doses and initiator concentration (**a**) 0%; (**b**) 0.1%; (**c**) 0.2% and (**d**) 0.3%.

The results of immersion experiments in distilled water to investigate the hydrogels’ behavior over time and to determine the values of swelling degree at equilibrium S_max_. (%) are shown in Figure 4 and Table 2.

The swelling degree is strongly influenced by the cross-linking degree, with the water absorption being inversely proportional to the cross-link density [44]. The cross-link density is, in turn, strongly influenced by the irradiation dose and the presence or absence of PP. In the absence of PP, the hydrogels obtained at 5 kGy showed a swelling degree of 34,423%, while at 20 kGy it was 5922%. This suggests that these hydrogels can incorporate into their structure an amount of water per gram of hydrogel of about 340 g/g and 60 g/g, respectively. A clear dependence between the swelling degree and irradiation dose, cross-linking degree (that increases from 0.044 × 10^2^ to 0.995 × 10^2^ mol/cm^3^), and mesh size (that decreases from about 220 nm to 26 nm) can be observed. The addition of only 0.1% PP led to hydrogels with a swelling degree of 42,954% (about 430 g/g) at the irradiation dose of 5 kGy and of 7206% (about 62 g/g) at 20 kGy, demonstrating higher swelling percentages than those obtained in the same irradiation conditions but without PP. Increasing PP concentration led to the obtaining of swelling degrees comparable with those obtained for samples without PP (except the irradiation doses of 20 kGy, where the swelling degree was even higher). Even though the cross-link density decreases with increasing PP concentration, at the 20 kGy irradiation dose, the mesh size also increases, possibly due to the degradative effects that compete with the cross-linking ones as a result of continuously generated free radicals [41]. Upon irradiation, a large number of free radicals and active sites [45] are formed throughout the system due to the decomposition of PEO [26,46,47], AA [48,49], and SA [48,49,50,51], mainly due to the water radiolysis process. These highly active free radicals created by high-energy irradiation initiate and drive the cross-linking and grafting reactions [45]. The slight decrease in swelling degree with increasing PP concentration at the irradiation dose of 5 kGy may be attributed to the self-crosslinking effect (bimolecular collision in the termination stage) [52,53]. In addition, the increasing PP concentration can lead to the degradation of the free radicals formed on the biopolymer chain by sulfate anion radicals, which is associated with a decrease in swelling [52].

Even if the hydrogels obtained at 5 kGy showed high swelling degrees, they showed, at the same time, fragility, after reaching S_max_ (Table 2).

**Figure 5:**
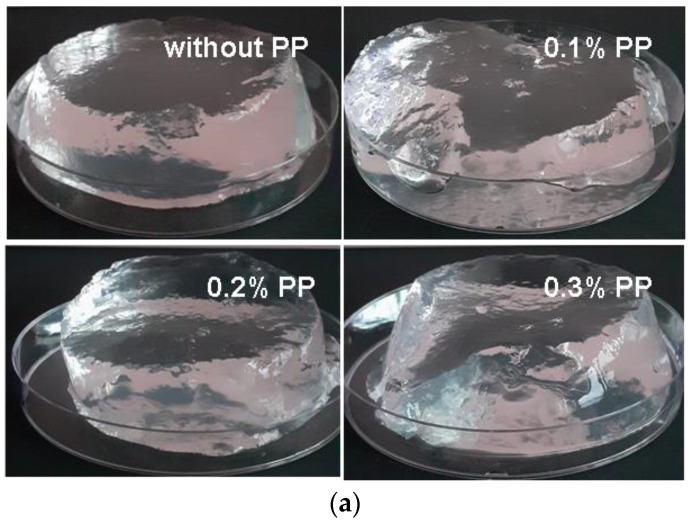

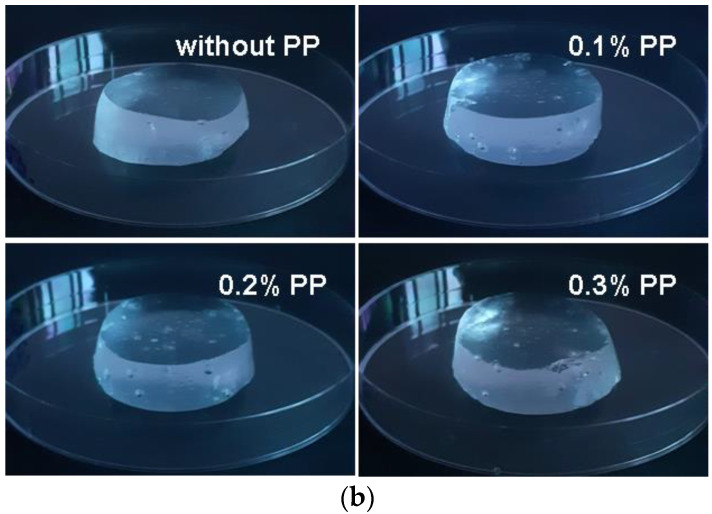
Images of hydrogels in the swollen state with and without PP and obtained at (**a**) 5 kGy, respectively (**b**) 20 kGy.

Figure 5 shows images of hydrogels obtained at 5 and 20 kGy. In the upper part of Figure 5a, the images of brittle hydrogels obtained at the irradiation dose of 5 kGy are exhibited, and in the lower part, the images of stabile hydrogels obtained at the irradiation dose of 20 kGy are also presented.

**Figure 6:**
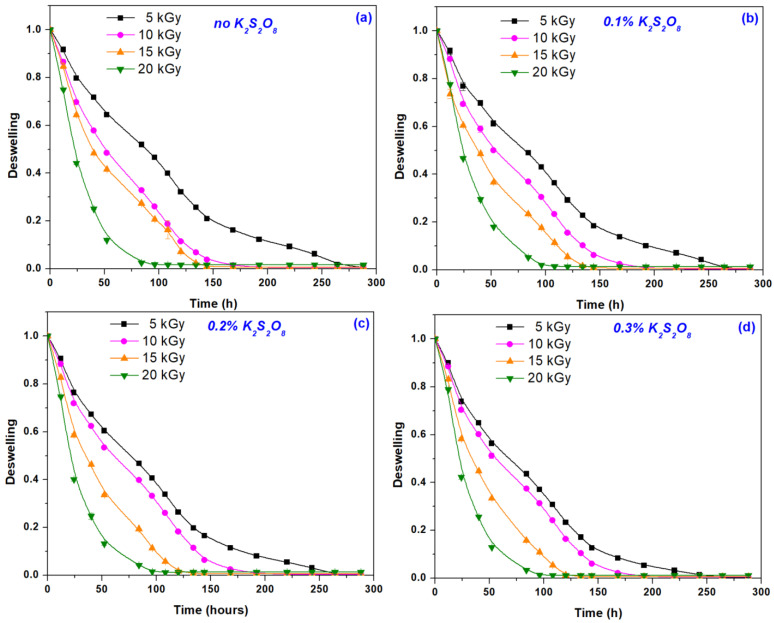
Deswelling degree in time of hydrogels immersed in distilled water depending on the irradiation doses and initiator concentration (**a**) 0%; (**b**) 0.1%; (**c**) 0.2% and (**d**) 0.3%.

In the experiments of deswelling kinetic, equilibrium-swelled hydrogels were placed in the air in laboratory dishes and weighed daily to constant mass. The deswelling curves are presented in Figure 6.

As can be seen from Figure 6, the degree of deswelling is influenced by the irradiation dose. Hydrogels obtained at the irradiation dose of 20 kGy lose water very quickly compared to those obtained at 5 kGy. By correlating this result with those obtained when determining the cross-linking degree, and mesh size, we can say that a high degree of cross-linking is associated with a small mesh size, and, consequently, the amount of water absorbed at equilibrium is small and its loss is rapid.

**Figure 7:**
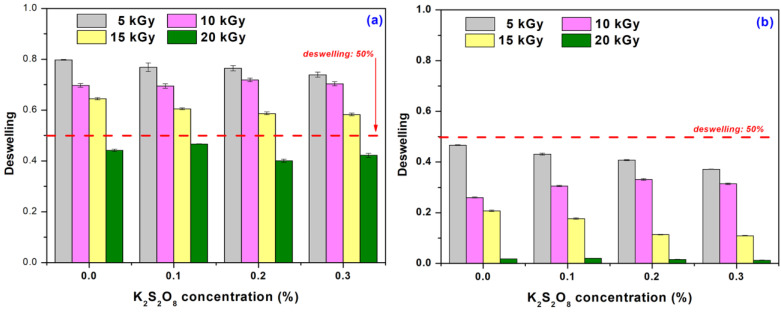
Deswelling degree of hydrogels immersed in distilled water depending on the irradiation doses and initiator concentration at (**a**) 24 h and (**b**) 96 h.

Figure 7 presents the influence of the initiator concentration on the hydrogels’ deswelling degree after 24 h and 96 h, respectively.

As can be seen from Figure 7, the hydrogels obtained at the irradiation dose of 15 kGy kept more than 60% of the water at room temperature for 1 day, the process varying very slightly with the increase in PP concentration. After 4 days, the water retention ratio is below 50% for the hydrogels obtained at 5 kGy, and with the increase in the irradiation dose up to 15 kGy, it drops even below 20%. The results are similar to those found in the literature [54].

The results presented so far up to this point complement other previously obtained studies [55] and show that the addition of PEO made it possible, at the same initiator concentration of 0.1%, to obtain hydrogels with improved properties at lower irradiation doses (S_max_, porosity, cross-link density).

### 3.2. Sol-Gel Analysis Results

The radiation parameters, p_0_/q_0_, Dg, and Dv, were calculated with the GelSol95 v1.0 software developed by the Division of Applied Radiation Chemistry at Lodz University of Technology [37,56]. The sol-gel parameters of hydrogels with different concentrations of PP are presented in Table 3. The p_0_/q_0_ values of hydrogels containing 0.1–0.3% PP were in the 0.20–0.34 range. If the p_0_/q_0_ parameter increases, the degradation occurs more efficiently than cross-linking. Generally, a higher value of the p_0_/q_0_ ratio indicates the presence of a degradation process [57].

The results showed a maximum value of 0.34 for hydrogels with 0.2% PP. The corresponding Dg, has decreased, while the K_2_S_2_O_8_ content has increased, except for the concentration of 0.2%. This behavior could suggest that the addition of a higher concentration of initiator could be responsible for the collapse of the radiation cross-linking reaction. This is due to the increasing number of polymeric chains and their derived radicals resulting in the final breakage of polymeric chains [58]. Moreover, above 0.1% PP, no mathematical correlation was found to confirm the evolution of the cross-linking process through irradiation.

The G_X_ values have varied between 1.80–12.60 × 10^−9^ mol/J and increased with the irradiation dose, being considerably higher than G_S_. The irradiation dose of 5 kGy showed the formation of hydrogels in which the G_X_ > G_S_ was almost dependent on PP concentration. Above 10 kGy, it is obvious that the cross-linking yields decrease with the increase of the initiator concentration. We can explain this behavior by the fact that the concentration increasing of generated free radicals has been higher as the irradiation dose and polymers concentrations have increased. In this case, the molecular weight increases continuously as a consequence of the cascade of generated free radicals. At some point, the increase in molecular weight stops, since the degradative effects will compete with the cross-linking effects due to the steric hindrance [41]. From another point of view, if the molecular weight will increase, the entanglement process will appear. Further, the entanglement will hinder the movement of the molecules and the radical recombination will be suppressed, inevitably leading to the appearance of degradative phenomena. In the materials having a G_S_:G_X_ ratio lower than the unit, the cross-linking has been favored, and oppositely, the degradation has been significant. As is shown in Table 4, the ratio Gs:Gx shows the increase in degradative effects as the percent of initiator is higher [59].

### 3.3. Structural Investigations Results (FTIR)

**Figure 8:**
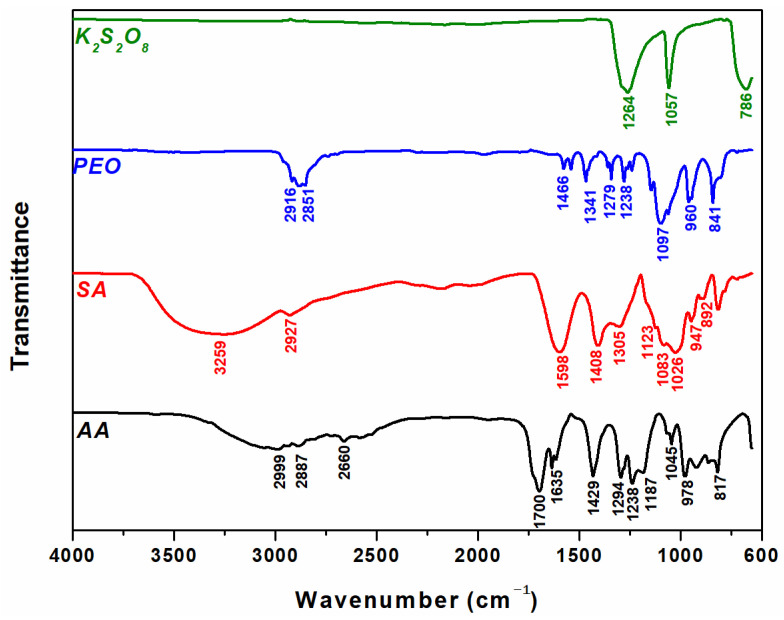
ATR-FTIR spectra of pure AA, SA, PEO and PP.

The ATR-FTIR spectra of pure SA, pure PEO, pure AA, and PP in the transmittance mode are presented in Figure 8.

The FTIR spectrum of pure SA shows a broad absorption band at 3259 cm^−1^ for the –OH stretching vibrations, the peak at 2927 cm^−1^ corresponding to the C–H stretching, the peak at 1598 cm^−1^ is assigned to C=O stretching, the peaks at 1408/1305/1123/1026/947 cm^−1^ correspond to the COO^−^ stretching, CH_2_ bending, C–O–C and C–O stretching [60].

In the spectra of PEO, the main peaks are: 2916/2851 cm^−1^ (CH_2_ stretching vibration), 1466/1341 cm^−1^ (CH^2^ deformation vibrations), 1097 cm^−1^ (C-O-C antisymmetric elongation), and 960/841 (CH_2_ rocking plane vibrations) [61]. The specific absorption bands corresponding to AA are 2997/2887 cm^−1^ (O–H stretching), 1697 cm^−1^ (C=O stretching vibration), 1635/1615 cm^−1^ (C=C), 1432 cm^−1^ (CH_2_), 1238/1184 cm^−1^ (C–O, and O–H of carboxylic groups), and 978/816 cm^−1^ (CH_2_ rocking mode) [62]. The FTIR spectrum of PP displays three large intense bands at 1264 cm^−1^, 1057 cm^−1^, and 768 cm^−1^ due to the S–O stretch, S=O asymmetric stretch, and symmetric stretch of sulfonic acid groups respectively [63].

**Figure 9:**
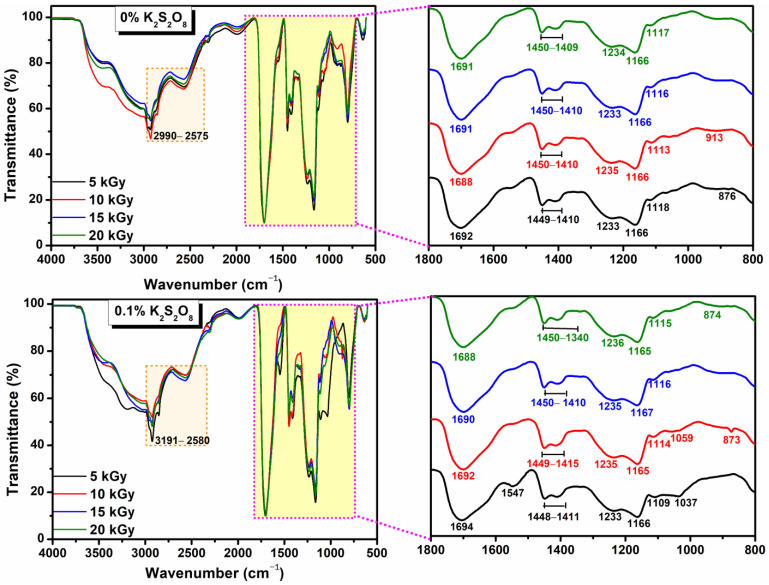
ATR-FTIR spectra of the cross-linked hydrogels with 0% respectively 0.1% K_2_S_2_O_8_.

Figure 9 shows the FTIR spectra of hydrogels containing different concentrations of K_2_S_2_O_8,_ prepared by electron beam irradiation at doses of 5–20 kGy.

According to the FTIR spectra of irradiated hydrogels without initiator (0% PP), in the 3000–2550 cm^−1^ range, the intensity of the bands decreases and the shift towards higher wave numbers depends on the irradiation dose. The strong absorption bands at 1688–1692 cm^−1^ specific to AA confirm the presence of the –C=O groups for all hydrogels. The bands in the 1409–1450 cm^−1^ range represent the bending vibration for the CH_2_ and CH_3_ groups and the band at 1233 cm^−1^ is due to the O–H bending vibration. The shifting in bands indicates the cross-linking between AA, PEO, and SA in the hydrogel. Only for the hydrogel with 0.1% PP cross-linked at 5 kGy, two new peaks are confirmed, at 3191 cm^−1^ and 1547 cm^−1^ corresponding to O–H stretching vibrations to SA and C=O stretching vibrations specific to AA and SA. Moreover, with the addition of PP, and after cross-linking of the hydrogels with 5 kGy and 10 kGy, the presence of the peak at 1057 cm^−1^ is attributed to sulfonic groups, indicating the strong interactions between the polymer components (SA, AA, PEO, and PP. The intensity of the absorption band depends on the concentration of the initiator used. The presence of PP, even small, affects the structure of the hydrogels, as seen from the swelling data and the average molar mass of the chain between cross-links.

### 3.4. Morphological Investigations Results (SEM)

**Figure 10:**
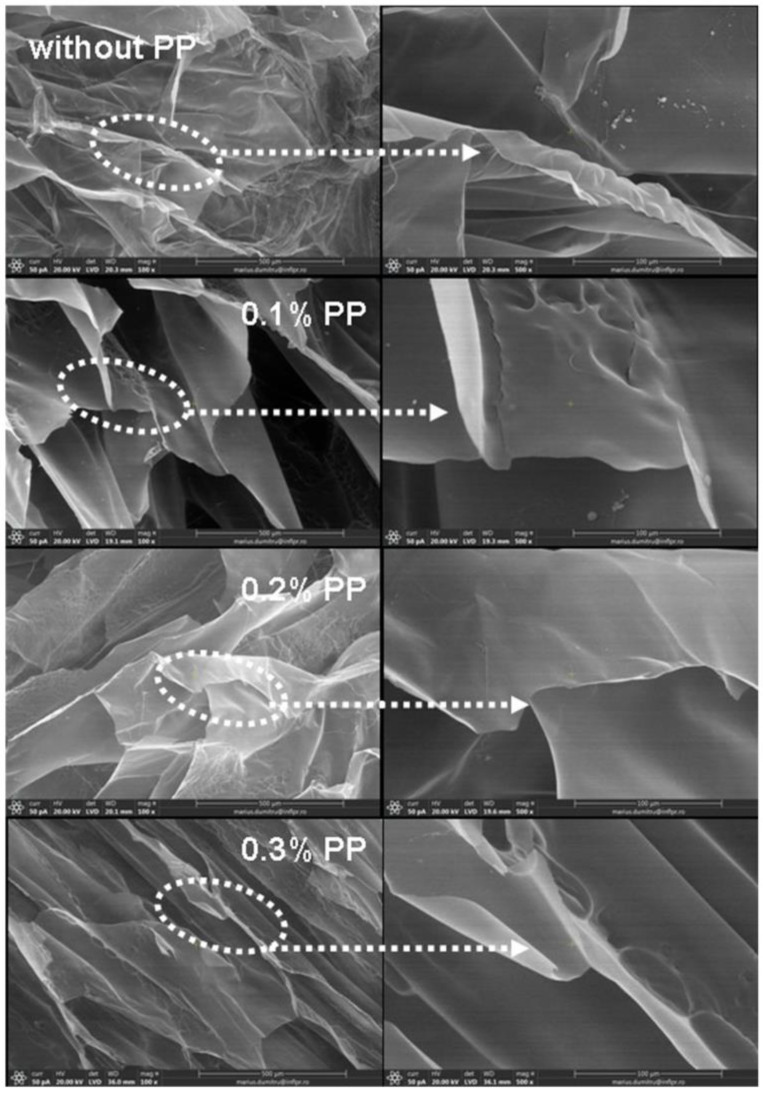
SEM micrographs of hydrogels were obtained at 5 kGy irradiation dose and at 100× (**left side**), 500× (**right side**) magnifications.

**Figure 11:**
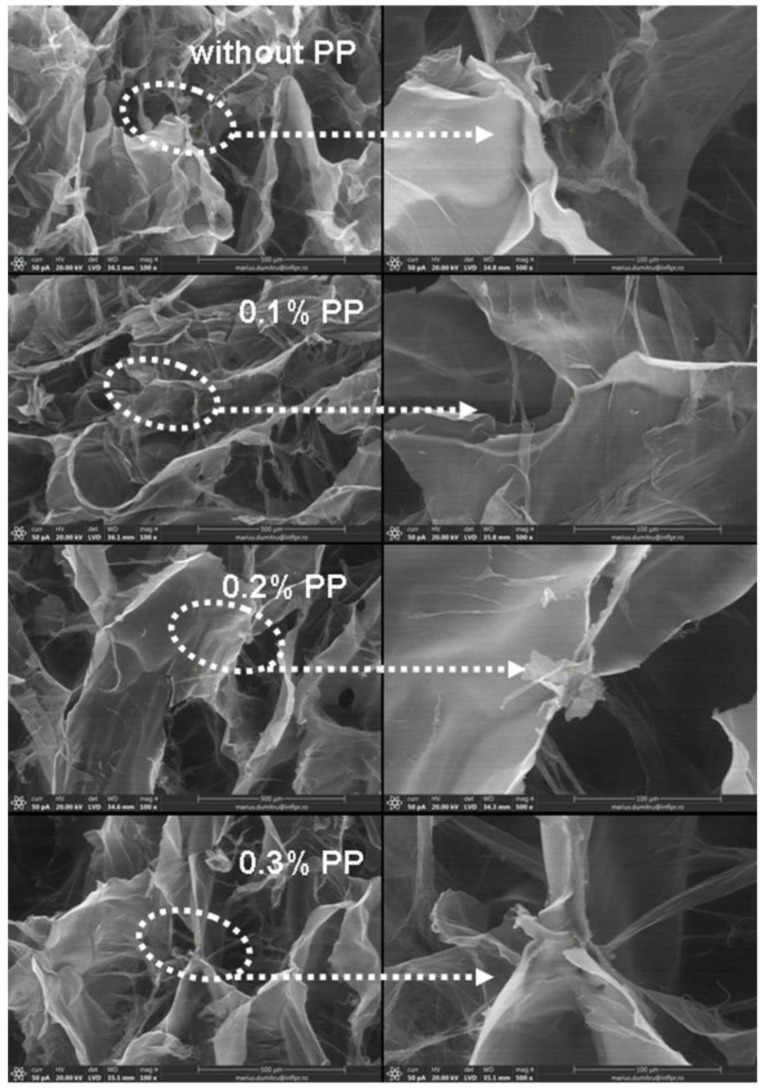
SEM micrographs of hydrogels were obtained at 20 kGy irradiation dose and 100× (**left side**), 500× (**right side**) magnifications.

The inner morphology of hydrogels in a freeze-drying state was investigated by Scanning Electron Microscopy (SEM). Micrographs of samples obtained at 5 kGy and 20 kGy, with and without PP, are presented in Figure 10 and Figure 11.

As can be seen from Figure 10, hydrogels obtained at 5 kGy have shown, in the inner structure, macropores with a slice-like aspect [64]. The decrease in pore size was very well correlated with the increase in initiator amount and reflected by the results obtained in swelling tests (swelling degree decreasing).

In Figure 11, the porous structure with interconnecting pores of hydrogels obtained at 20 kGy can be observed [65]. The pores are small but regular, with homogeneous, thin walls. At the irradiation dose at which the cross-linking and degradation processes compete, at 20 kGy, the homogeneity of the structure with stronger cross-links was still observed in some places.

## 4. Conclusions

Hydrogels based on acrylic acid (AA), sodium alginate (SA), and poly(ethylene oxide) (PEO) were obtained by electron beam irradiation using potassium persulfate (PP) as the reaction initiator. Additionally, the influence of initiator concentration and irradiation dose on hydrogel network parameters, swelling properties, gelation and degradation points, structure, and morphology were investigated.

Gel fraction, cross-link density, and mesh sizes are affected by both irradiation dose and initiator concentration. Because grafting and cross-linking reactions occur even at low irradiation doses, gel fractions over 87% have been found in hydrogels obtained at the smallest irradiation dose of 5 kGy. Increasing the irradiation dose leads to an increase in gel fraction to 94.5%. The addition of 0.3% PP has led to a slight decrease in the gel fraction, up to 92.1% at the irradiation dose of 20 kGy. The concentration of 0.1% PP at irradiation doses of 5 and 10 kGy has led to the obtaining of stabilized hydrogels in the swollen state, characterized by gel fraction, cross-link densities, and mesh sizes appropriate to the intended purpose of obtaining them. Thus, swelling degrees over 40,000% have been obtained using hydrogels with 0.1% PP irradiated at 5 kGy. Hydrogels with swelling degrees around 30,000% were obtained at the same irradiation dose of 5 kGy without PP but also with 0.2 and 0.3% PP. In all other analyzed situations (irradiation doses of over 5 kGy correlated with the absence or with each of the three concentrations of PP), swelling degrees were around 20,000%.

The processes of AA and SA cross-linking, grafting of AA on SA and PEO chains, and degradation of SA have been investigated and highlighted by FTIR analysis. It has been found that the irradiation dose of 20 kGy facilitates the SA degradation due to the breakage of the main chains of alginate and the presence of PP accelerates the same process at the same time with increasing irradiation dose.

## Figures and Tables

**Table 1:** The values of the average molar mass of the chain between cross-links, M_c_, the polymer volume fraction in the swollen gel, *υ*_2*,s*_ × 10^3^, and porosity, P, of hydrogels.

Irradiation Dose (kGy)	PP, K_2_S_2_O_8_, Concentration (%)			
	0	0.1	0.2	0.3
M_c_ (kg/mol)				
**5**	719.0 ± 52.7	825.0 ± 42.8	545.8 ± 35.7	348.1 ± 21.7
**10**	375.6 ± 30.5	294.6 ± 31.6	273.0 ± 18.9	296.0 ± 27.0
**15**	160.8 ± 14.9	109.2 ± 7.9	137.9 ± 12.9	147.2 ± 11.9
**20**	31.9 ± 3.0	48.2 ± 5.9	45.7 ± 4.0	54.2 ± 4.6
*υ*_2*,s*_ × 10^3^ (cm^3^)				
**5**	2.1 ± 0.09	1.9 ± 0.06	2.4 ± 0.09	3.0 ± 0.11
**10**	3.3 ± 0.16	3.4 ± 0.22	3.5 ± 0.14	3.5 ± 0.19
**15**	5.2 ± 0.28	6.5 ± 0.28	5.6 ± 0.31	5.4 ± 0.26
**20**	13.4 ± 0.73	10.5 ± 0.76	10.8 ± 0.56	9.8 ± 0.49
P (%)				
**5**	99.7 ± 0.01	99.8 ± 0.01	99.7 ± 0.01	99.7 ± 0.01
**10**	99.5 ± 0.02	99.6 ± 0.03	99.6 ± 0.02	99.5 ± 0.03
**15**	99.2 ± 0.04	99.2 ± 0.03	99.3 ± 0.04	99.3 ± 0.03
**20**	98.3 ± 0.09	98.6 ± 0.10	98.6 ± 0.07	98.8 ± 0.06

**Table 2:** The values of swelling degree at equilibrium S_max_ (%).

Irradiation Dose (kGy)	PP (K_2_S_2_O_8_) Concentration (%)			
	0	0.1	0.2	0.3
**5**	34,423 ± 1509	42,954 ± 1333	32,854 ± 1285	29,130 ± 1088
**10**	20,469 ± 994	23,197 ± 1490	24,338 ± 1004	21,331 ± 1163
**15**	13,189 ± 729	13,105 ± 712	13,960 ± 779	14,879 ± 717
**20**	5922 ± 330	7206 ± 528	7360 ± 387	8040 ± 408

**Table 3:** The sol-gel parameters for hydrogels with different concentrations of K_2_S_2_O_8_.

K_2_S_2_O_8_	p_0_/q_0_	D_g_ (kGy)	D_v_ (kGy)	R^2^
0%	0.20	1.20	−0.52	0.92
0.1%	0.27	0.97	−0.51	0.99
0.2%	0.34	2.96	−2.90	-
0.3%	0.32	0.59	0	-

**Table 4:** The radiation-chemical yield of cross-linking (G_X_) and scission (G_S_) was calculated for hydrogels.

K_2_S_2_O_8_ (%)	G_X_, G_S_ × 10^−9^ mol/J				G_S_:G_X_ (20 kGy)
	5 kGy	10 kGy	15 kGy	20 kGy	
0	2.04 ^1^/0.83 ^2^	1.80 ^1^/0.73 ^2^	2.85 ^1^/1.16 ^2^	12.60 ^1^/5.15 ^2^	0.40
0.1	1.95 ^1^/1.08 ^2^	3.17 ^1^/1.75 ^2^	5.23 ^1^/2.89 ^2^	8.01 ^1^/4.42 ^2^	0.55
0.2	2.92 ^1^/2.01 ^2^	3.14 ^1^/2.16 ^2^	3.89 ^1^/2.68 ^2^	8.94 ^1^/6.16 ^2^	0.68
0.3	5.06 ^1^/3.29 ^2^	2.60 ^1^/1.69 ^2^	3.75 ^1^/2.44 ^2^	7.47 ^1^/4.56 ^2^	0.61

^1^—G_X_; ^2^—G_S_.

## Data Availability

Data sharing is not applicable to this article.
